# Maternal type 1 diabetes mellitus and atopic dermatitis in offspring: A nationwide cohort study

**DOI:** 10.1016/j.jdin.2026.03.013

**Published:** 2026-03-30

**Authors:** Sarah Weissmann, Nir Amitai, Eliya Honig, Tamar Eshkoli, Amir Horev

**Affiliations:** a Faculty of Health Sciences, Ben-Gurion University of the Negev, Beer Sheva, Israel; b Clinical Research Unit, Soroka University Medical Center, Beer Sheva, Israel; c Department of Obstetrics and Gynecology, Soroka University Medical Center, Beer Sheva, Israel; d Pediatric Dermatology Service, Soroka University Medical Center, Beer Sheva, Israel

**Keywords:** atopic dermatitis, diabetes mellitus, eczema, maternal type 1 diabetes, national data

## Abstract

**Background:**

Atopic dermatitis (AD) is one of the most common chronic inflammatory skin diseases of childhood and contributes substantially to early-life morbidity. Maternal metabolic and allergic conditions have been linked to offspring atopic disease; however, the effect of maternal type 1 diabetes mellitus (T1DM), a chronic autoimmune condition, on AD risk in offspring has not been well defined.

**Objectives:**

To examine the association between maternal T1DM during pregnancy and the prevalence of atopic dermatitis in offspring.

**Methods:**

We conducted a nationwide retrospective cohort study using electronic medical records from Clalit Health Services, Israel’s largest health maintenance organization. Singleton live births between January 1, 2010, and December 31, 2023, were included, with follow-up through December 31, 2024. Maternal T1DM and offspring AD were identified using International Classification of Diseases, 10th Revision codes. Multivariable logistic regression models were used to estimate associations, adjusting for maternal age, socioeconomic status, smoking status, gestational age, infant sex, delivery mode, ethnicity, allergic rhinitis, and follow-up duration.

**Results:**

The cohort included 311,708 mother–child pairs, of whom 1104 (0.35%) were born to mothers with T1DM. Atopic dermatitis was more prevalent among offspring of mothers with T1DM compared with those without T1DM (29% vs 26%). In adjusted analyses, maternal T1DM was associated with increased odds of AD in offspring (odds ratio 1.15; 95% confidence interval 1.00-1.32). Additional independent predictors included male sex, maternal smoking, maternal allergic rhinitis, higher socioeconomic status, and shorter gestational age. Maternal hemoglobin A1c levels >6.5% were not associated with AD prevalence.

**Conclusions:**

In this large population-based cohort, maternal T1DM was associated with an increase in the prevalence of AD in offspring. While the magnitude of the effect is small, these findings suggest that maternal autoimmune status may contribute to early-life atopic risk.

**Capsule Summary**

• Maternal metabolic and allergic conditions, including gestational diabetes, obesity, and atopy have been associated with increased risk of atopic disease in offspring.
• Using nationwide longitudinal data, we show that children born to mothers with type 1 diabetes have a higher risk of atopic dermatitis independent of maternal, perinatal, and socioeconomic factors, highlighting the importance of maternal metabolic and immune function in early life atopic conditions.

## Introduction

Atopic dermatitis (AD) is the most common chronic inflammatory skin disease and is characterized by recurrent eczematous lesions and intense pruritus.^1^^,^^2^ AD usually presents in infancy and has been shown to affect up to 30% of children in developed countries. AD has a high global burden and can severely impact quality of life through itch, depression, sleep disturbance, and anxiety.^3^ Asthma, allergic rhinitis, and food allergies are often seen in patients with AD.^1^ Though the pathophysiology of AD is not completely understood, the current understanding is that an impaired skin barrier causes individuals to be more susceptible to xerosis and environmental irritants and allergens, which subsequently trigger inflammation via a type 2 dominated response.^4^

Due to the high prevalence and burden of AD in children, maternal factors that may contribute to the development of AD have been investigated. Previous studies have identified several risk factors for AD, including maternal gestational diabetes (GDM), maternal history of atopy, exposure to stress, antibiotics, and smoking.^5^, ^6^, ^7^, ^8^ Maternal GDM and status as large for gestational age have been associated with wheezing and asthma, and large for gestational age has been associated with atopic sensitization, serum IgE, and AD.^6^^,^^9^, ^10^, ^11^, ^12^, ^13^, ^14^, ^15^, ^16^, ^17^, ^18^, ^19^, ^20^, ^21^, ^22^, ^23^ Although the impact of large for gestational age and GDM on AD has been investigated, to our knowledge no study has investigated the impact of maternal Type 1 diabetes (T1DM) on early childhood development of AD. Given the autoimmune nature and metabolic influence of T1DM, we hypothesized that maternal T1DM is associated with an increased prevalence of AD in offspring.

## Methods

### Data collection

This retrospective cohort study was conducted using the Clalit Health Services (CHS) data sharing platform, powered by MDClone (https://www.mdclone.com). This platform utilizes advanced algorithms to deidentify and extract data from electronic medical records, ensuring both data integrity and patient privacy. The study was approved by the local Ethics Committee of Soroka University Medical Center (No. 0049-25-SOR).

### Study population

This study utilized electronic medical and birth records from Clalit Health Services (CHS), the largest health maintenance organization in Israel, serving approximately 4.8 million individuals, accounting for 51% of the national population. The cohort included births occurring between January 1, 2010, and December 31, 2023. Follow-up data were available through the end of 2024, and the inclusion period was restricted to births up to 2023 to ensure a minimum follow-up duration of 1 year for all participants.

Inclusion criteria comprised records of live births among infants born during the study period to mothers concurrently enrolled in Clalit Health Services (CHS). The initial dataset included 331,025 birth records. Exclusion of 19,317 births resulting from multiple gestations yielded a final analytic cohort of 311,708 singleton infants.

### Variable definitions

The main exposure variable was maternal T1DM during pregnancy, identified using relevant ICD-10 diagnosis codes recorded prior to delivery. The primary outcome was the prevalence of AD in offspring, defined according to ICD-10 codes extracted from the CHS database (L20.9). Diagnoses were included regardless of whether they were made during hospitalization or outpatient visits.

Additional maternal and perinatal covariates included maternal age at delivery, parity, gestational age, mode of delivery, ethnicity, socioeconomic status (SES), maternal allergic rhinitis, maternal AD, maternal smoking status, child sex and follow-up duration. Maternal glycated hemoglobin (HbA1C) levels, measured between 6 and 18 months prior to delivery, were also collected as an indicator of glycemic control and a proxy for disease severity. SES was determined based on a municipality-level index developed by the Israeli Central Bureau of Statistics, incorporating 14 socioeconomic indicators. Municipalities were ranked on a decile scale (1-10) and subsequently grouped into 3 categories: low (1-3), medium (4-6), and high (7-10).

### Statistical analysis

Descriptive statistics were used to characterize the study population, including baseline demographic and clinical variables. Continuous variables are presented as means with standard deviations (SDs) or medians with interquartile ranges, while categorical variables are summarized as frequencies and percentages. Comparisons of continuous variables were performed using the *t*-test for normally distributed data and the Mann–Whitney *U* test for non-normally distributed data. Categorical variables were compared using the Chi-square test or Fisher's exact test when expected cell counts were low.

The association between maternal T1DM and AD in offspring was assessed using logistic regression to estimate odds ratios (ORs) and 95% confidence intervals (CIs), comparing infants born to mothers with T1DM to those without. Multivariable models were adjusted for maternal age, SES, gestational age at birth, child birth weight and sex, child asthma and allergic rhinitis, maternal atopic dermatitis, follow-up duration and maternal smoking status.

To account for variable follow-up duration among children and to evaluate the timing of atopic dermatitis onset, we additionally performed a time-to-event analysis using a Cox proportional hazards regression model. Time-to-event was defined as the interval from birth to the first recorded diagnosis of atopic dermatitis, with children censored at the end of follow-up or at the time of leaving the Clalit Health Services database. Both crude and multivariable Cox models were estimated. The multivariable model adjusted for the same covariates included in the primary logistic regression analysis, including maternal age, socioeconomic status, gestational age, child sex, maternal smoking status, maternal allergic rhinitis, maternal atopic dermatitis, and follow-up duration. Hazard ratios (HRs) and 95% confidence intervals (CIs) were calculated. Results of the Cox proportional hazards analysis are presented in Supplementary Table I, available via Mendeley at https://data.mendeley.com/datasets/x3z79thx7w/1.

All statistical tests were two-tailed, with a significance level set at *P* < .05. Analyses were conducted using R software (version 4.2.3).

## Results

A total of 311,708 mother-child pairs were included in the cohort. Of these, 1104 children (0.35%) were born to mothers with T1DM (Table I). Mothers with T1DM were slightly older at delivery (mean 31.8 vs 30.1 years, *P* < .001), had lower parity (1.39 vs 1.66, *P* < .001), delivered at an earlier gestational age (37.1 vs 38.9, *P <* .001), and were more likely to deliver preterm (22% vs 5.6%, *P* < .001) and by cesarean section (48% vs 17%, *P* < .001) compared with mothers without T1DM. Significant differences were also observed across socioeconomic status; a higher percentage of T1DM mothers in a medium socioeconomic group (58% vs 50%, *P <* .001). There was no statistically significant difference between smoking status of mothers (11% vs 9.2%, *P* = .082). Mothers with T1DM did not have higher prevalence of allergic rhinitis, AD or other atopic states compared to mothers without T1DM.

**Table I:** Patient characteristics of mothers with T1DM compared to mothers without T1DM

Characteristic	Overall,*N* = 311,708∗	Mothers withoutT1DM, *N* = 310,604	Mothers withT1DM, *N* = 1104	*P* value†
Maternal age at delivery				<.001
Mean ± SD (*N*)	30.1 ± 5.5 (311,708)	30.1 ± 5.5 (310,604)	31.8 ± 5.3 (1104)	
Median (IQR)	30.1 (26.0, 34.1)	30.1 (26.0, 34.1)	31.7 (27.8, 35.5)	
Range	15.2, 50.0	15.2, 50.0	19.1, 46.8	
Maternal parity, mean ± SD (*N*)	1.66 ± 1.76 (311,700)	1.66 ± 1.76 (310,596)	1.39 ± 1.51 (1104)	<.001
Gestational wk				<.001
Mean ± SD (*N*)	38.92 ± 1.72 (311,532)	38.93 ± 1.71 (310,429)	37.12 ± 1.73 (1103)	
Median (IQR)	39.00 (38.00, 40.00)	39.00 (38.00, 40.00)	38.00 (37.00, 38.00)	
Range	24.00, 44.00	24.00, 44.00	28.00, 41.00	
Preterm delivery, *n* (%)	17,739 (5.7%)	17,501 (5.6%)	238 (22%)	<.001
Delivery type, *n* (%)				<.001
Cesarean	45,547 (17%)	45,057 (17%)	490 (48%)	
Vaginal	228,165 (83%)	227,636 (83%)	529 (52%)	
Smoking status, *n* (%)				.082
Nonsmoker	278,363 (90%)	277,398 (90%)	965 (88%)	
Past smoker	3709 (1.2%)	3694 (1.2%)	15 (1.4%)	
Smoker	28,562 (9.2%)	28,440 (9.2%)	122 (11%)	
Ethnicity, *n* (%)				<.001
Arabic	34,591 (11%)	34,479 (11%)	112 (11%)	
Bedouin	62,158 (20%)	62,063 (20%)	95 (8.9%)	
Jewish	210,051 (68%)	209,195 (68%)	856 (81%)	
Socioeconomic score, *n* (%)				<.001
Low	87,919 (32%)	87,692 (32%)	227 (22%)	
Medium	140,369 (50%)	139,775 (50%)	594 (58%)	
High	49,894 (18%)	49,694 (18%)	200 (20%)	
Allergic rhinitis, *n* (%)	40,439 (13%)	40,309 (13%)	130 (12%)	.2
Atopic dermatitis, *n* (%)	26,557 (8.5%)	26,458 (8.5%)	99 (9.0%)	.6
Other atopic state, *n* (%)	61,055 (20%)	60,843 (20%)	212 (19%)	.7
Range	1.0, 14.0	1.0, 14.0	1.0, 14.0	
Child gender, *n* (%)				.4
Female	151,547 (49%)	150,995 (49%)	552 (50%)	
Male	160,158 (51%)	159,606 (51%)	552 (50%)	

*T2DM*, Type 2 diabetes mellitus.

∗ Mean ± SD (*N*); *n* (%).

† Wilcoxon rank sum test; Pearson’s Chi-squared test.

Table II shows the prevalence of AD in offspring of mothers with and without T1DM. Offspring of mothers with T1DM had a higher prevalence of AD compared with offspring of mothers without T1DM (29% vs 26%, *P* = .006). The median age at diagnosis of AD did not differ significantly between groups (1.28 vs 1.27 years, *P* > .9).

**Table II:** Offspring AD of mothers with T1DM compared to mothers without T1DM

Characteristic	Overall, *N* = 311,708∗	Mothers without T1DM, *N* = 310,604	Mothers with T1DM, *N* = 1104	*P* value†
Offspring atopic dermatitis, *n* (%)	79,953 (26%)	79,630 (26%)	323 (29%)	.006
Offspring atopic dermatitis age at diagnosis				>.9
Mean ± SD (*N*)	1.99 ± 2.12 (79,953)	1.99 ± 2.12 (79,630)	1.89 ± 1.92 (323)	
Median (IQR)	1.27 (0.51, 2.57)	1.27 (0.51, 2.57)	1.28 (0.57, 2.45)	
Range	0.01, 14.96	0.01, 14.96	0.09, 9.33	

*T1DM*, Type 1 diabetes mellitus.

∗ *n* (%)

† Fisher’s exact test; Wilcoxon rank sum test; Pearson’s Chi-squared test.

Maternal HbA1c >6.5% was not associated with overall prevalence of AD (29% vs 28%, *P* = .6), although age at diagnosis was slightly later in this subgroup (median 1.30 vs 1.12 years, *P* = .010), as seen in Table III.

**Table III:** Offspring morbidities by HbA1C >6.5

Characteristic	Overall, *N* = 31,641∗	FALSE, *N* = 30,874	TRUE, *N* = 767	*P* value†
Offspring atopic dermatitis, *n* (%)	8836 (28%)	8615 (28%)	221 (29%)	.6
Offspring atopic dermatitis age at diagnosis				.010
Mean ± SD (*N*)	1.74 ± 1.86 (8836)	1.73 ± 1.86 (8615)	1.97 ± 1.88 (221)	
Median (IQR)	1.13 (0.47, 2.21)	1.12 (0.47, 2.20)	1.30 (0.65, 2.68)	
Range	0.02, 14.10	0.02, 14.10	0.09, 9.25	

∗ *n* (%).

† Fisher’s exact test; Wilcoxon rank sum test; Pearson’s Chi-squared test; Wilcoxon rank sum exact test.

In Table IV, multivariable logistic regression adjusting for smoking status, child birth weight, ethnicity, delivery type, allergic rhinitis, child gender, maternal age, socioeconomic score, child follow-up duration, and gestational week, maternal T1DM was associated with the presence of AD in offspring (OR 1.15, 95% CI 1.00-1.32, *P* = .045). Additional predictors of AD included parental smoking, male sex, coexisting allergic rhinitis, higher socioeconomic status, and shorter gestational age.

**Table IV:** Multivariate logistic regression

	Crude			Adjusted		
	OR	95% CI	*P*-value	OR	95% CI	*P* value
Any DM diagnosis	1.20	1.05, 1.37	.007	1.15	1.00, 1.32	.051
Smoking						
Nonsmoker				—	—	
Past smoker				1.23	1.14, 1.32	<.001
Smoker				1.13	1.10, 1.16	<.001
Child birth weight						
Allergic rhinitis				1.26	1.23, 1.29	<.001
Child gender						
Female				—	—	
Male				1.09	1.07, 1.11	<.001
Maternal age at delivery				1.00	1.0, 1.00	<.001
Socioeconomic score						
Low				—	—	
Medium				1.87	1.83, 1.91	<.001
High				2.18	2.12, 2.24	<.001
Child follow-up duration				1.03	1.03, 1.03	<.001
Gestational wk				1.03	1.02, 1.03	<.001
Maternal asthma				1.13	1.10, 1.17	<.001
Maternal atopic dermatitis				1.32	1.29, 1.36	<.001

*DM*, Diabetes mellitus.

To account for differences in follow-up duration and evaluate the timing of AD onset, a Cox proportional hazards analysis was performed (Supplementary Table I, available via Mendeley at https://data.mendeley.com/datasets/x3z79thx7w/1). In the adjusted model, maternal T1DM was not significantly associated with time to AD diagnosis in offspring (HR 1.10, 95% CI 0.98-1.24, *P* = .093). Male sex, maternal allergic rhinitis, and greater gestational age were associated with a higher hazard of AD diagnosis, whereas higher socioeconomic status was associated with a slightly lower hazard. Overall, the results of the time-to-event analysis were consistent in direction with the primary logistic regression analysis but did not reach statistical significance.

## Discussion

In this large, population-based cohort study, we found that maternal T1DM was associated with a modest increase in the prevalence of AD in offspring. Children born to mothers with T1DM had a 3% higher prevalence of AD than children born to nondiabetic mothers (29% vs 26%), and this association remained significant after adjustment for maternal, perinatal, and socioeconomic factors. Although statistically significant, the magnitude of this association was small and may have limited clinical significance at the population level. To our knowledge, this is the first study to directly evaluate the impact of maternal T1DM on early childhood AD.

Our findings extend prior research on maternal metabolic conditions and allergic disease. Previous studies have shown that GDM is associated with an increased risk of childhood AD and allergen sensitization.^6^^,^^9^, ^10^, ^11^, ^12^, ^13^, ^14^, ^15^, ^16^, ^17^, ^18^, ^19^, ^20^, ^21^, ^22^, ^23^ These studies suggest that maternal dysglycemia may influence fetal immune development and skin barrier function through in utero metabolic and inflammatory exposures.^24^ Alternatively, it has been proposed that increased levels of cytokines, adipokines, and oxidative stress mediators impact the developing immune system causing dysregulation.^24^ It is also possible that changes in the maternal-fetal environment may favor a Th2-skewed response in the offspring contributing to atopic sensitization and AD.^24^

Another proposed mechanism is diabetes-associated changes to the maternal gut and vaginal microbiome, which may influence early microbial colonization and immune development in offspring. Pregnant women with T1DM have shown compositional and functional changes in the gut microbiome, including an increase in lipopolysaccharide producing bacteria and a decrease in short-chain fatty acid producing bacteria.^25^ In addition, pregnant women with T1DM have elevated levels of fecal calprotectin and other markers of intestinal damage and inflammation.^25^ These microbiome changes may be transmitted to the offspring during pregnancy, delivery, and early dietary and environmental exposures, potentially impairing immune tolerance and skin barrier maturation. Disrupted microbial exposure and low intestinal bacterial diversity in early life and in utero have been linked to increased prevalence of allergic diseases in childhood, including atopic dermatitis.^26^, ^27^, ^28^

The observed association between maternal T1DM and AD in offspring may be partly explained by shared autoimmune mechanisms. Recent research has found several similarities between AD and autoimmune conditions, including increased prevalence of IgE autoantibodies and antinuclear antibodies.^29^ Further, AD is associated with an increased prevalence of comorbid autoimmune diseases such as psoriatic arthritis, Sjögren syndrome, Crohn’s disease, vitiligo, alopecia areata, pernicious anemia, ulcerative colitis, rheumatoid arthritis, and hypothyroidism.^30^ Additionally, genetic factors such as cytokine and immune-related genes have been implicated in AD and have been linked to other autoimmune and inflammatory diseases as well, including psoriasis, Crohn’s disease, and asthma.^31^ It is possible that maternal autoimmunity during pregnancy could influence the developing fetal immune system through transplacental transfer of autoantibodies, shared genetic susceptibility, or epigenetic modifications, predisposing the child to AD.

Importantly, maternal atopic conditions were not more prevalent among mothers with T1DM, suggesting that the observed association may not be mediated by maternal atopic predisposition. Instead, the autoimmune mechanisms specific to T1DM, such as systemic inflammation, autoantibody transfer, or shared immune-regulatory pathways, may play a role independent of maternal atopic status.

Maternal HbA1c >6.5% was not associated with increased AD prevalence. Though these findings may point to autoimmune activity, genetic susceptibility or systemic inflammation as key mechanisms rather than maternal glucose control, previous studies have found gestational diabetes mellitus to be a risk factor for AD in offspring.^20^, ^21^, ^22^, ^23^ Our results, however, must be interpreted cautiously as HbA1c measurements obtained 6-18 months prior to delivery may not accurately reflect glycemic control during critical periods of fetal immune development.

Several predictors of AD were found in our multivariable analysis, consistent with previous studies. Male sex, maternal smoking, allergic rhinitis, and higher socioeconomic status were each associated with an increased risk of AD. Longer gestation was associated with a slightly increased risk of AD. Although previous literature is conflicting, recent publications have also shown that preterm birth is inversely associated with the risk of AD.^32^, ^33^, ^34^ It has been suggested that the fetal immune response to specific antigens strengthens with gestational age, and the intestinal nonimmune defense system matures, decreasing the risk of AD in full term infants.^35^^,^^36^

Although we performed a supplementary time-to-event analysis using a Cox proportional hazards model to account for variable follow-up duration, the association between maternal T1DM and AD did not reach statistical significance in this model, suggesting that the observed association may be modest.

These findings should not be interpreted as supporting immediate clinical interventions. Rather, maternal T1DM may represent one of several markers that in combination with other risk factors such as maternal atopy and genetic susceptibility, could help identify higher-risk populations in future research.

The strengths of this study include its large sample size, population-based design, and detailed clinical and demographic data, which allowed for adjustment of multiple potential confounders. However, several limitations should be acknowledged. First, AD diagnoses were based on ICD-10 coding without independent validation or severity assessment, raising the possibility of misclassification bias. Second, while we adjusted for socioeconomic and ethnic background, we were unable to account for several potentially important confounders, including paternal atopic history, breastfeeding, early-life antibiotic exposure, environmental factors, and family autoimmune history. Residual confounding from these variables may partially explain the observed association. Third, the number of T1DM mothers, while substantial for this rare condition, was small relative to the total cohort, which may limit the statistical power of our analyses.

In conclusion, we demonstrate that maternal T1DM is independently associated with increased risk of AD in offspring, extending previous findings on gestational diabetes and allergic disease. These results highlight the potential importance of maternal metabolic and immune function in early-life atopic conditions and underscore the need for further research to clarify mechanisms, identify higher-risk subgroups, and determine whether targeted preventative strategies are warranted. Future studies should explore underlying mechanisms, including the role of autoimmunity, chronic inflammation, and epigenetic modifications, and should evaluate whether better glycemic control before and during pregnancy could mitigate this risk.

## Conflicts of interest

None disclosed.
